# Clinical Prevalence of Equine Coital Exanthema in a Thoroughbred Covering Station in Türkiye (2021–2024)

**DOI:** 10.1111/rda.70086

**Published:** 2025-06-09

**Authors:** Yunus Emre Atay, Gencay Ekinci, Ali Erdem Öztürk, Mustafa Cem Timur, Alper Mete, Köksal Altınbay, Fatih Mehmet Derelli, Yaşar Akar, İhsan Keleş

**Affiliations:** ^1^ Faculty of Veterinary Medicine, Department of Obstetrics and Gynecology Erciyes University Kayseri Türkiye; ^2^ Faculty of Veterinary Medicine, Department of Internal Medicine Erciyes University Kayseri Türkiye; ^3^ Faculty of Veterinary Medicine, Department of Reproduction and Artificial Insemination Erciyes University Kayseri Türkiye; ^4^ Equine Health and Veterinary Services, Head Office Jockey Club of Türkiye İstanbul Türkiye; ^5^ Jockey Club of Türkiye İstanbul Equine Hospital İstanbul Türkiye; ^6^ Jockey Club of Türkiye Izmit Central Covering Station Kocaeli Türkiye

**Keywords:** clinical prevalence, equine coital exanthema, equine herpesvirus type 3, molecular diagnosis

## Abstract

Equine Coital Exanthema (ECE) is an endemic herpesvirus disease primarily affecting the external genitalia and impairing mating activities in horses. Its extremely contagious nature, latency and subclinical features can result in outbreaks and significant economic losses. Transmission occurs primarily through mating activities; therefore, robust biosecurity measures are crucial in breeding facilities. This study aims to determine the clinical prevalence of ECE among horses in a covering station in Türkiye from 2021 to 2024. It also aims to assess the efficacy of routine PCR implementation within ECE's control strategies. A cross‐sectional study design has been employed. Genital swab samples were collected from clinically suspected horses, which were tested for EHV‐3 using real‐time PCR. Animal records, clinical examination data and PCR test results were obtained from horses at the covering station between 2021 and 2024. During the 4 years (2021–2024), 9231 mating activities were carried out, and a total of 228 clinically suspected horses were tested for EHV‐3 using real‐time PCR. Among these 228 horses, 6 horses (2.6%) were confirmed positive for EHV‐3. The primary weakness of this study is the failure to detect subclinical cases with PCR. The absence of follow‐up PCR testing in two clinically infected horses represents a limitation of this study. The molecular diagnosis of ECE was reported for the first time in Türkiye. Clinical ECE cases infrequently transpired over the four‐year period at the covering station. No outbreak transpired during this interval. PCR testing plays a crucial role in disease control when implemented with suitable management methods. Additional global epidemiological investigations on ECE are required.

## 1 Introduction

Equine coital exanthema (ECE) is an external genital infection caused by equine herpesvirus type 3 (EHV‐3) (Barrandeguy and Thiry 2012). It is clinically characterised by painful lesions such as papules, vesicles, pustules and ulcers that develop on the genital areas of horses. The disease typically heals within 10–14 days; however, it poses potential challenges to the equine industry by disrupting mating activities and reducing reproductive efficiency (Barrandeguy, Vissani, et al. 2010; Vissani et al. 2021) The virus induces latency similar to other herpesviruses, allowing horses to remain asymptomatic carriers for prolonged durations. Viral transmission primarily occurs through coitus. Additionally, non‐coital routes such as contaminated fomites and close behavioural interactions (e.g., genital–nasal interaction) also contribute to disease spread. Despite previous suggestions, there is no scientific evidence supporting mechanical transmission by stable flies or through urine (Vissani et al. 2021; Barrandeguy, Perkins, et al. 2010; Ataseven 2013).

ECE was first documented in Ireland in the early 1900s (Craig and Kehoe 1921). Equine herpesvirus type 3 was first isolated concurrently in the United States, Canada and Australia in 1968 (Pascoe et al. 1969; Girard et al. 1968; Bryans 1968). Today, it is endemic worldwide, with seroprevalence rates varying across populations: 4.1% in Japan (Kirisawa et al. 2017), 22.9% in Mongolia (Pagamjav et al. 2011) and 48% in Argentina (Vissani et al. 2021). In Türkiye, there is a paucity of data regarding the prevalence of ECE. A prior seroprevalence investigation conducted by Ataseven et al. (2014) indicated rates of 51.2% in brood horses, 10.2% in racehorses and 9.3% in working horses. Nevertheless, no molecular investigations have been undertaken to validate ECE in Türkiye. Therefore, this study was conducted to evaluate the occurrence of ECE at a covering station in Türkiye during 2021–2024. This study employs molecular diagnosis via real‐time PCR, marking the inaugural report of ECE's prevalence in Türkiye. These findings seek to address deficiencies in disease surveillance and aid in formulating global strategies for the management and control of an endemic disease.

## 2 Materials and Methods

### 2.1 Ethics Committee

Ethical approval was obtained from the Erciyes University Animal Experiments Local Ethics Committee under protocol number 24/185.

### 2.2 Data Source

This study was conducted using animal records, clinical examination data and PCR test results from the İzmit Central Covering Station between 2021 and 2024. The station is a semi‐closed type, operated by the Jockey Club of Türkiye in İzmit, Türkiye. This establishment is among the largest equine breeding operations in the country. The station employs a team of veterinarians, technicians, laboratory staff and grooms to ensure optimal health and productivity of the horses. The facility operates under strict biosecurity protocols to prevent the spread of infectious diseases, including regular PCR screening for ECE in horses clinically suspected of infection. Since 2023, these tests have been conducted in its own laboratory; during 2021 and 2022, PCR testing was carried out through a service purchase from Veterinary Control Central Research Institute, Ankara, Turkey.

### 2.3 Clinical Examination Procedures

All mares and stallions were inspected for external genitalia and rectal examinations were performed routinely during pre‐mating clinical examinations. Horses displaying clinical signs, such as vesicles, pustules, or ulcers on the external genital organs, were suspected of ECE. Additionally, horses with non‐specific symptoms on the genital organs, such as vaginal discharge and vulvar hyperaemia, were also sampled. Suspected cases were defined through molecular diagnosis using real‐time PCR targeting the glycoprotein G gene of EHV‐3. Stallions underwent first and second follow‐up PCR testing on Days 7–10 and Days 14–15 post‐diagnosis, respectively, while in mares, follow‐up PCR testing was scheduled only on Days 14–15. However, additional testing at weekly intervals was considered if the previous follow‐up PCR result was positive.

### 2.4 Sampling and Nucleic Acid Isolation

Sterile swabs were used to collect samples from the vulvar region of symptomatic mares and the penis of stallions. Samples were placed in a sterile tube containing 1 mL of sterile isotonic saline solution and immediately transferred to the laboratory. Samples were centrifuged at 1500 rpm for 5 min. The supernatant (300 μL) was used for nucleic acid isolation, performed with the TanBead Maelstrom 4810 system (Taiwan Advanced Nanotech Inc., Taiwan) using the viral nucleic acid isolation kit (TanBead Optiure Viral Auto Tube) according to the manufacturer's protocol.

### 2.5 Real‐Time PCR Analyses

PCR analyses were performed using TaqMan fluorogenic probe‐based quantitative PCR to detect the glycoprotein G gene according to Vissani et al. (2018) (Table 1) The reaction mixture was prepared with 10 μL of qPCR master mix, 400 nM primers, 200 nM probe and nuclease‐free sterile distilled water. Amplification was performed in the real‐time PCR system (LongGene Q2000B, LongGene Europe Ltd., Türkiye) under the following conditions: 95°C for 10 min, followed by 40 cycles of 95°C for 30 s and 60°C for 30 s. Cycle threshold (Ct) values below 35 were interpreted as positive, while values above 35 were negative.

**TABLE 1:** Primer sequences used for the detection of EHV‐3.

Name	Primers	Target	Reference
EHV‐3 Fw	GGGTATCGGCTTTCTCATCTTG	*gG* gene	Vissani et al. (2018)
EHV‐3 Rv	CCGCAGGACGCAAACG	*gG* gene	
EHV‐3 Probe	6‐FAM‐TGTGTCTCCTCATCGGCCTCATTGTCT‐TAMRA	*gG* gene	

Abbreviations: Fw, forward; gG, glycoprotein G; Rv, reverse.

### 2.6 Statistical Analyses

Statistical analyses were performed using the IBM‐SPSS for Windows Release 25.0 Program (SPSS Inc., Chicago, IL, USA). Data were expressed as % (*n*/total). The relationship between categorical variables was evaluated using Pearson's 2 test (and Fisher's exact test). A *p* value < 0.05 was considered statistically significant.

## 3 Results

During the study period (2021–2024), a total of 228 clinically suspected horses were tested for EHV‐3 using real‐time PCR. Among these, 6 horses (2.6%) were confirmed positive for EHV‐3. The annual distribution of positive cases, tested horses and the number of total mating activities is summarised in Table 2.

**TABLE 2:** Annual distribution of positive cases and number of mating activities.

Year	Matings	Stallions	Mares	Tested horses	Positive cases	Prevalence in tested (%)
2021	1967	20	1181	26	1	3.8
2022	2048	20	1313	31	2	6.5
2023	2706	20	1647	9	0	0.0
2024	2510	20	1559	162	3	1.9
Total	9231	80	5700	228	6	2.6

Overall, the prevalence of EHV‐3 among tested horses remained low across the years, with the highest positivity rate observed in 2022 (6.5%). Despite minor annual fluctuations, the prevalence did not exhibit a statistically significant trend (*p* > 0.05) (Table 3).

**TABLE 3:** Distribution and comparison of EHV‐3 positive cases over the years.

Years	EHV‐3 (+)	EHV‐3 (−)	χ^2^
	% (*n*/total)	% (*n*/total)	*p*
2021	3.8 (1/26)	96.2 (25/26)	2.543 0.480
2022	6.5 (2/31)	93.5 (29/31)	
2023	0 (0/9)	100 (9/9)	
2024	1.9 (3/162)	98.1 (159/162)	

Among the horses tested positive for EHV‐3, one was identified in 2021, two in 2022 and three in 2024. There were no EHV‐3‐positive horses in 2023. All EHV‐3‐positive horses were Thoroughbreds.

Out of the six positive horses, two of them were stallions (33.3%), and four were mares (66.7%). Their median age was determined to be 7 years (range 5–12).

The present study found no statistically significant correlation between age and gender categories (*p* = 0.600, *χ* ^2^ = 3.000). Two mares and one stallion were aged 3–6 years, whereas the other two mares were aged 7–9 years (Table 4).

**TABLE 4:** Prevalence of equine herpesvirus 3 by age class.

Age (years)	Mare	Stallion	χ^2^
	EHV‐3 (*n* = 4)	EHV‐3 (*n* = 2)	*p*
3–6	50.0 (2/4)	50.0 (1/2)	3.000 0.600
7–9	50.0 (2/4)	0 (0/2)	
10 >	16.7 (0/4)	50.0 (1/2)	

*Note:* Data were expressed as % (*n*/total).

Among the six EHV‐3 positive horses, two remained positive on Days 7–10, while four were not tested. By Days 14–15, four tests were negative while two were not tested (Table 5). The Ct values of PCR‐positive samples are presented in Table 6.

**TABLE 5:** Test results of EHV‐3 cases.

Horse no	Year	Sex	Test results		
			Day 0	Days 7–10	Days 14–15
1	2021	Mare	Positive	np	np
2	2022	Stallion	Positive	Positive	Negative
3	2022	Mare	Positive	np	Negative
4	2024	Stallion	Positive	Positive	Negative
5	2024	Mare	Positive	np	np
6	2024	Mare	Positive	np	Negative

Abbreviation: np, not performed.

**TABLE 6:** Ct values of PCR‐positive samples.

Horse No	Year	Sex	Ct values	
			Day 0	Days 7–10
1	2021	Mare	26.54	np
2	2022	Stallion	18.72	29.15
3	2022	Mare	30.59	np
4	2024	Stallion	23.58	28.33
5	2024	Mare	30.08	np
6	2024	Mare	18.20	np

Abbreviation: np, not performed.

### 3.1 Clinical Observations

**FIGURE 1:**
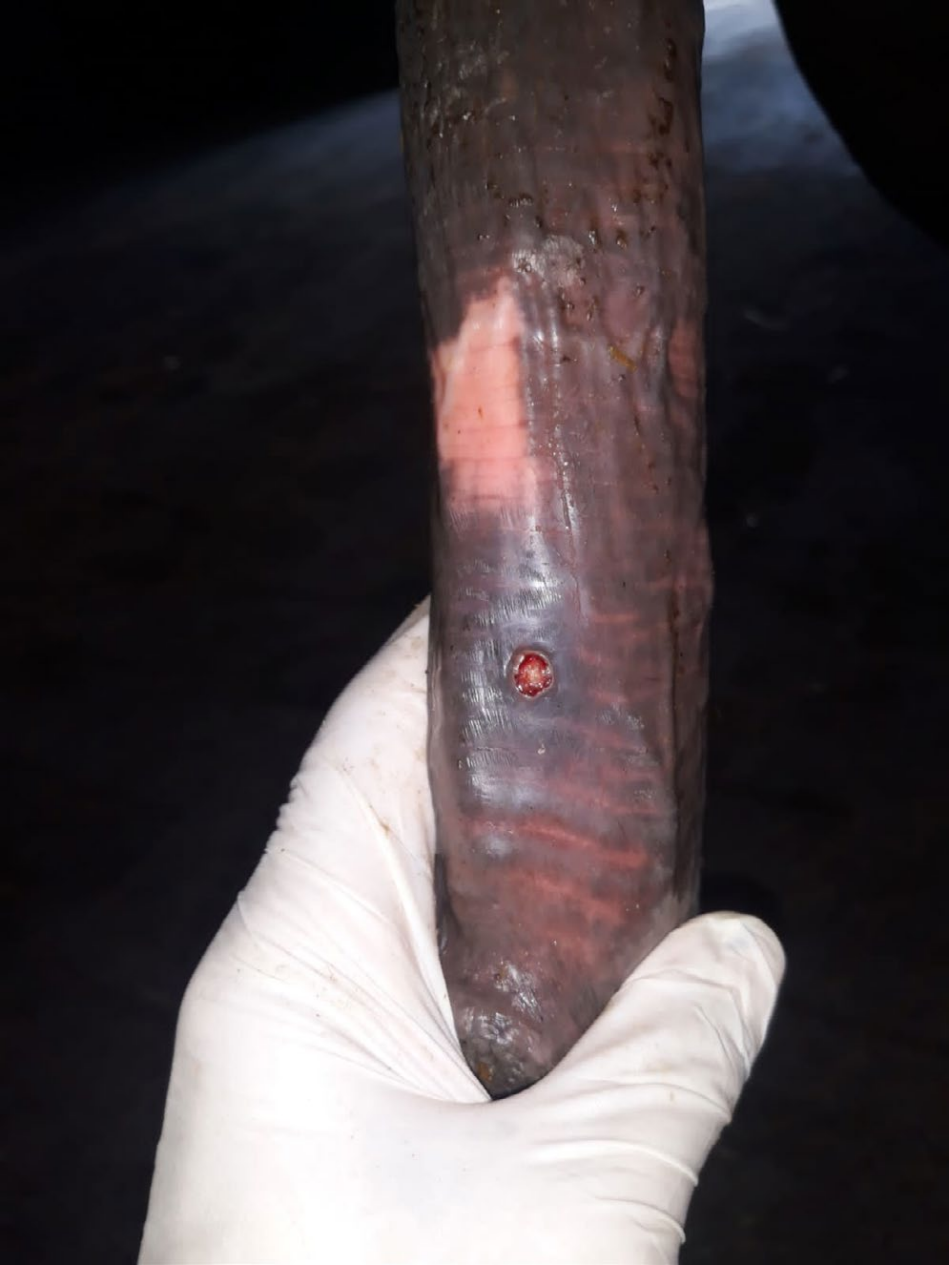
Typical crater‐like lesion on the penile body.

**FIGURE 2:**
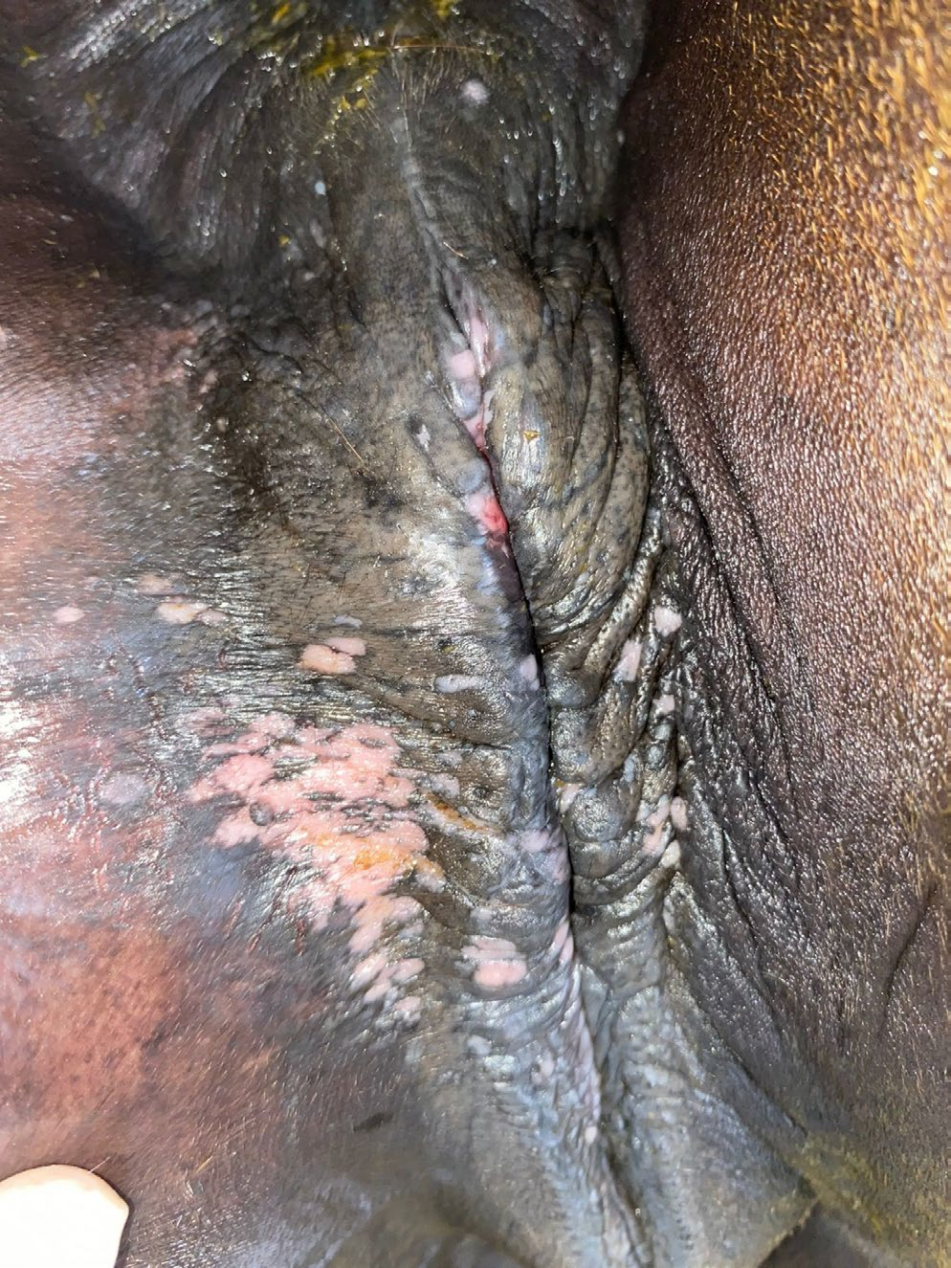
Lesions on the vulva during healing and leaving white plaque‐like scars.

All PCR‐positive horses (*n* = 6) exhibited clinical signs consistent with equine coital exanthema (ECE), including vesicular and ulcerative lesions localised to the external genitalia (Figures 1, 2). No subclinical or asymptomatic carriers were tested among the population.

### 3.2 Real‐Time PCR Results

**FIGURE 3:**
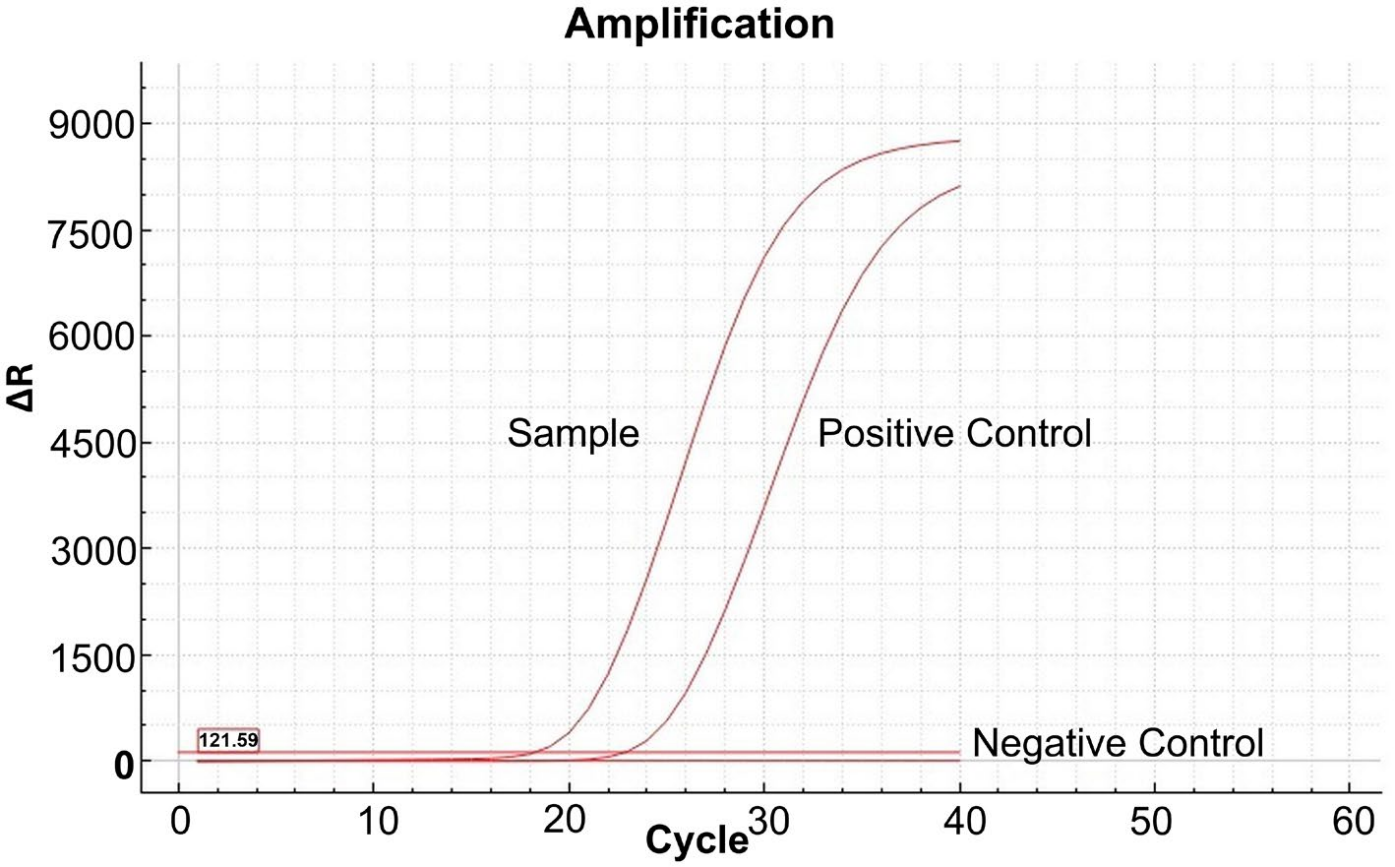
Real‐time PCR amplification plot for EHV‐3. The sample showed a Ct value of 18.2, while the positive control amplified at Ct 22.8. No amplification was observed in the negative control (nuclease‐free water), confirming the validity of the assay.

Real‐time PCR analyses successfully amplified the glycoprotein G gene of EHV‐3 in all positive cases. Negative controls consistently yielded Ct values > 35, ensuring the specificity of the assay (Figure 3).

## 4 Discussion

Equine coital exanthema (ECE), caused by equine herpesvirus‐3 (EHV‐3), has a significant economic impact due to the temporary disruption of mating activities (Barrandeguy, Vissani, et al. 2010). In a study conducted in Türkiye, seroprevalence rates of ECE were reported as 51.2% in breeding horses, 10.2% in racehorses and 9.3% in working horses (Ataseven et al. 2014). This research represents the inaugural molecular detection of EHV‐3 within a substantial semi‐closed stud population at a covering station in İzmit/Türkiye. In the current study, EHV‐3 was detected in 6 out of 228 horses suspected of having ECE between 2021 and 2024, which corresponds to an overall rate of 2.6%. A retrospective study conducted in France reported that, between 2010 and 2021, EHV‐3 was found to be positive in 16 out of 71 suspected cases, indicating a rate of 22% (Hue et al. 2021). In another study, EHV‐3 was detected in 14 out of 220 perineal‐vaginal swab samples taken from asymptomatic thoroughbred mares, which corresponds to a rate of 6% (Barrandeguy, Vissani, et al. 2010). Unlike our study, which was conducted in a semi‐closed breeding population with regular veterinary monitoring, the study by Hue et al. (2021) analysed field samples collected nationwide over a 12‐year period. While case definition criteria were not clearly provided in their abstract, contextual differences in sample collection, population exposure and surveillance intensity may explain the discrepancy in EHV‐3 detection rates.

Clinical signs in this viral infection develop after an incubation period of 4–7 days, and these lesions typically heal within 10–14 days if cases are uncomplicated with other pathogens (Paccamonti and Crabtree 2019). Systemic signs such as dullness, anorexia and fever may rarely occur (Brinsko et al. 2011; Ley and Slusher 2007). Non‐genital lesions are also rarely observed on the skin, mucous membranes of the respiratory tract and lips (Żychska et al. 2024). In the present study, following the literature (Żychska et al. 2024), papules, vesicles, erosions and ulcerative lesions were detected on the external genital organs of mares and stallions (Figures 1, 2).

In the current study, although the number of horses tested in 2024 (*n* = 162) was higher than in previous years (26 horses in 2021, 31 horses in 2022 and 9 horses in 2023), the number of EHV‐3 positive cases was comparable to those identified in prior years. Consistent with the findings of Hue et al. (2021) the annual case numbers over a four‐year period at a station where a total of 9231 matings occurred ranged from 0 to 3. Despite the observation of positive cases fluctuating between 0 and 3 in this semi‐closed population with high mating activity, an outbreak did not occur. This can be attributed to the well‐implemented disease control strategies at the station. These control strategies include clinical examinations, biosecurity measures, hygiene procedures before and after mating, laboratory testing and isolation of infected horses (Vissani et al. 2021; Vissani 2024). In this covering station, different PCR monitoring strategies were implemented for stallions and mares due to their respective roles in the breeding programme. Stallions were closely monitored and tested at approximately one‐week intervals to allow their prompt return to mating activities without disrupting the breeding schedule. For mares, however, follow‐up PCR testing was planned around days 14–15, as re‐mating would not occur until the next estrous period. This approach aimed to confirm virological recovery at a clinically appropriate time point while minimising unnecessary testing during the early post‐diagnosis phase for mares. However, due to logistical and technical limitations related to laboratory access, follow‐up PCR testing could not be performed on two of the six horses at days 14–15, as originally planned. Although these horses had shown clinical recovery, the lack of virological confirmation may pose a minor biosecurity risk, as subclinical or prolonged viral shedding cannot be completely ruled out. The absence of follow‐up PCR testing in two horses represents a limitation of this study.

Despite conflicting results regarding the prevalence of EHV‐3 in stallions and mares (Kirisawa et al. 2017; Toishi et al. 2017), it is generally expressed that EHV‐3 is more commonly observed in mares (Vissani 2024; Seki et al. 2004; Troncoso et al. 2023). A study conducted in Türkiye found a higher seropositivity rate in mares compared to stallions (Ataseven et al. 2014). Żychska et al. (2024) reported that a total of 11 mares and 4 stallions were clinically diagnosed during cases and outbreaks that occurred at four different times. In a study by Seki et al. (2004), it was noted that 2 stallions and 18 mares were affected by ECE in working horses in Japan. In the current study, among the 6 horses that tested positive for EHV‐3, 4 (66.7%) were mares and only 2 (33.3%) were stallions. This observation can be explained by the higher number of mares compared to stallions at the station. Indeed, at the covering station, there were between 1181 and 1647 mares but only 20 stallions annually.

In the current study, EHV‐3 was detected in both mares and stallions. However, the exact source of transmission to other horses at the station remains unclear. It has been emphasised that ECE can also spread through mechanical means, such as infected materials and veterinary equipment, especially in an embryo transfer centre where natural mating does not occur (Barrandeguy, Perkins, et al. 2010). Therefore, mechanical transmission sources should always be considered in breeding stations. Additionally, another significant reason for the transmission between horses could be subclinical cases. The latent nature of EHV‐3, like other herpesviruses, plays an important role in its epidemiology. The reactivation of latent cases can serve as a viral source, whether clinically apparent or subclinical (Vissani et al. 2021). In a study conducted in Argentina, the virus was detected in 6% of mares without clinical signs (Barrandeguy, Vissani, et al. 2010). The virus can occasionally be isolated from external genital culture sites in subclinical cases, which means a potentially high risk of virus spread from clinically healthy horses (Barrandeguy, Vissani, et al. 2010). Disease may recur as a result of stimuli such as stress, systemic disease, or genital area trauma (Barrandeguy and Thiry 2012; Barrandeguy et al. 2008). Due to their frequent interactions with numerous mares and their elevated workload and stress levels during the breeding season, stallions are at a heightened risk of contracting EHV‐3 (Barrandeguy and Thiry 2012; Toishi et al. 2017). Additionally, a subclinical stallion has the potential to cause an outbreak by infecting a large number of mares during the breeding season. One important limitation of this study is that subclinical infected horses were not specifically investigated, which may have led to an incomplete understanding of the extent and epidemiological dynamics of EHV‐3 circulation. To prevent the subclinical spread of the virus among breeding horses, it has been recommended as a biosecurity protocol to perform on‐site real‐time PCR to segregate them from mating (Vissani et al. 2020). Furthermore, it has been reported that iiPCR, as a rapid diagnostic tool, offers a significant advantage in field conditions by demonstrating a high accuracy rate when used in conjunction with real‐time PCR (Vissani et al. 2018).

## 5 Conclusion

In this study, the molecular prevalence of ECE‐suspected horses was revealed for the first time in a covering station in Türkiye in a four‐year period. Despite the presence of clinical infections, an outbreak has not occurred during this period. Prevention‐based management practices, including PCR testing, hold a significant role in the disease control strategies. When appropriate control strategies are implemented, including PCR testing of suspected cases, it is possible to prevent outbreaks as in this study. However, PCR‐based diagnosis of subclinical cases may be required for controlling the disease in high‐risk facilities. Further epidemiological studies on ECE are needed globally. To better monitor the disease course and ensure complete resolution, the use of PCR should be extended beyond initial diagnosis to include regular follow‐up testing. Only after virological recovery has been confirmed should animals be reintegrated into the breeding program.

## Author Contributions

Conceptualisation: Y.E.A., G.E., M.C.T., F.M.D.; Methodology: Y.E.A., G.E., M.C.T., F.M.D.; Investigation: Y.E.A., G.E., M.C.T., A.M., K.A., F.M.D.; Data curation: M.C.T., A.M., K.A., F.M.D.; Formal analysis: Y.E.A., G.E., A.E.Ö., Y.A., İ.K.; Writing – original draft: Y.E.A., G.E., A.E.Ö.; Writing – review and editing: Y.E.A., G.E., Y.A., İ.K.; Supervision: Y.A., İ.K.

## Conflicts of Interest

The authors declare no conflicts of interest.

## Data Availability

The data that support the findings of this study are available from the corresponding author upon reasonable request.
